# Processing of the SARS-CoV pp1a/ab nsp7–10 region

**DOI:** 10.1042/BCJ20200029

**Published:** 2020-03-13

**Authors:** Boris Krichel, Sven Falke, Rolf Hilgenfeld, Lars Redecke, Charlotte Uetrecht

**Affiliations:** 1 Heinrich Pette Institute, Leibniz Institute for Experimental Virology, Martinistraße 52, 20251 Hamburg, Germany; 2 University of Hamburg, Institut für Biochemie und Molekularbiologie, Martin-Luther-King-Platz 6, 20146 Hamburg, Germany; 3 University of Lübeck, Institute of Biochemistry, Center for Structural and Cell Biology in Medicine, Ratzeburger Allee 160, 23562 Lübeck, Germany; 4 German Center for Infection Research (DZIF), Hamburg-Lübeck-Borstel-Riems Site, University of Lübeck, Ratzeburger Allee 160, 23562 Lübeck, Germany; 5 Deutsches Elektronen Synchrotron (DESY), Notkestraße 85, 22607 Hamburg, Germany; 6 European XFEL GmbH, Holzkoppel 4, 22869 Schenefeld, Germany

**Keywords:** coronavirus, native mass spectrometry, polyprotein processing, structural virology

## Abstract

Severe acute respiratory syndrome coronavirus is the causative agent of a respiratory disease with a high case fatality rate. During the formation of the coronaviral replication/transcription complex, essential steps include processing of the conserved polyprotein nsp7–10 region by the main protease M^pro^ and subsequent complex formation of the released nsp's. Here, we analyzed processing of the coronavirus nsp7–10 region using native mass spectrometry showing consumption of substrate, rise and fall of intermediate products and complexation. Importantly, there is a clear order of cleavage efficiencies, which is influenced by the polyprotein tertiary structure. Furthermore, the predominant product is an nsp7+8(2 : 2) hetero-tetramer with nsp8 scaffold. In conclusion, native MS, opposed to other methods, can expose the processing dynamics of viral polyproteins and the landscape of protein interactions in one set of experiments. Thereby, new insights into protein interactions, essential for generation of viral progeny, were provided, with relevance for development of antivirals.

## Introduction

The discovery of severe acute respiratory syndrome coronavirus (SARS-CoV) as the causative agent of the SARS epidemic in 2003 brought the world's attention to the human pathogenic potential of zoonotic infections by coronaviruses [1,2]. Two-thirds of their single-strand (+)-sense RNA genome exhibit the overlapping replicase genes ORF 1a and ORF 1ab, and the other one third encode for a set of subgenomic mRNAs which are required for accessory proteins as well as for the structural proteins. Initially, ORF 1a and ORF 1ab are directly translated into either replicase polyprotein pp1a (nsp1–11) or pp1ab (nsp1–16), respectively, depending on a ribosomal (-1)-frameshift [3]. Subsequently, the polyproteins undergo proteolytic processing into 11 or 16 individual nsp's. Eventually, the nsp's take part in forming the replication/transcription complex (RTC), a membrane-anchored, highly dynamic protein–RNA complex facilitating the replicative processes [4–7].

Two CoV proteases facilitate the processing of the polyprotein, the papain-like protease (PL^pro^; nsp3) between nsp1–4 and main chymotrypsin-like protease (M^pro^; 3CL^pro^, nsp5) between nsp4–11/16. Due to their essential roles, these proteases were extensively investigated regarding their interplay of structure and function, and their suitability as drug target [8–11].

The activity of M^pro^ is regulated over mechanisms comprising maturation from the polyprotein by autoprocessing of nsp4–5 and nsp5–6, as well as concentration and substrate induced self-assembly into an active dimeric unit [12–14]. After freeing itself, M^pro^ liberates nsp's 6–16 from the polyprotein by specifically targeting their nsp inter-domain junctions. These primarily contain LQ↓S or LQ↓A residues at the positions P2, P1 and P1′, but the strictly conserved requirement is only Q at P1 [15].

Temporal and spatial coordination of polyprotein processing is a way to regulate specific functions in the life-cycle of +ssRNA viruses [16,17]. The coordinated processing of pp1a is crucial for replication [18]. This was shown in detail for the processing order of the nsp7–10 region, where domain deletions or switching as well as cleavage site mutations were lethal to the virus replication [19]. In general, the order of processing is directly dependent on the substrate specificity of M^pro^, which has been tested in peptide-based biochemical assays [20–22]. However, peptide assays interrogate merely the substrate specificity for the isolated amino-acid sequence but disregard the polyprotein's structural layout, i.e. overall conformation or accessibility.

Upon processing of the nsp7–10 region by M^pro^, the four small proteins nsp7, nsp8, nsp9 and nsp10 are released [23]. They can form functional complexes with CoV core enzymes and thereby stimulate replication. Most importantly, a complex of nsp7+8 acts as a processivity factor for nsp12, the RNA-dependent RNA-polymerase (RdRp) [24]. Furthermore, point mutations in SARS-CoV nsp7 and nsp8 delayed virus growth or were identified as critical for virus replication [24]. Particularly, various oligomeric states of nsp7+8 have been reported for SARS-CoV and Feline infectious peritonitis virus (FIPV) [25–29]. Therefore, the interaction of nsp7 and nsp8 appears to be conserved across the coronavirus family making these proteins interesting drug targets, which highlights the need of understanding their structural flexibility.

Detailed studies of polyprotein processing and complex formation require a method that follows these dynamic processes at a molecular level. Native mass spectrometry (MS) probes and detects protein complexes in protein mixtures, while keeping their non-covalent interactions intact through nano-electrospray ionization (nanoESI) [30,31]. Furthermore, collision-induced dissociation (CID) enables the stoichiometry and topology of natively sprayed protein complexes to be deciphered, by dissociating non-covalent interactions *in vacuo*. Thereby, it allows the landscape of protein species from a heterogeneous sample solution to be revealed and to observe dynamic processes over time. Herein, we analyzed the processing of SARS-CoV nsp7–10 and complex formation of its sequentially released nsp's. First, the substrate specificity of SARS-CoV M^pro^ for the cleavage sites within nsp7–10 was determined in an assay with FRET-labeled peptides. Then, we explored native MS to reveal time-resolved nsp release and complex formation upon processing of a full-length SARS-CoV nsp7–10 substrate. Specifically, the analysis via CID-MS gave insights into a preferred complex of SARS-CoV nsp7+8.

## Materials and methods

### Cloning and gene constructs

To generate inserts for expression plasmids for the CoV ORF1ab nsp7–10 region, DNA was amplified by PCR from commercially obtained cDNA (Eurofins scientific SE) and from DNA plasmids existing from earlier work [23,27]. To create ends for directed cloning, the PCR products were digested by Eco31I (BsaI) (Thermo Fisher Scientific) and ligated into IBA pASK33+ and pASK35+, encoding for C- and N-terminal His-tag, respectively. The expression plasmid for SARS-CoV M^pro^ with authentic ends was created as described by Xue et al. [32].

### Expression and purification

Main protease SARS-CoV M^pro^ was produced with authentic ends [32]. Briefly, the protein constructs had a GST-tag connected via an auto-cleavage site at the N-terminus and a His-tag connected via a PreScission protease cleavage site at the C-terminus. The protease was purified by affinity chromatography using Ni^2+^-NTA beads (Thermo Fisher Scientific) and dialyzed into storage buffer (50 mM HEPES, 10% glycerol pH 7.5). The His-tag was cleaved by dialysis into PreScission cleavage buffer (20 mM Tris–HCl, 150 mM NaCl, 1 mM EDTA, 1 mM DTT at pH 7.0) while incubating with PreScission protease (protease to protein ratio 1 : 2000) (GE Healthcare) overnight. PreScission protease was removed by binding to GST-Sepharose (GE Healthcare) for 2 h. Finally, M^pro^ was buffer exchanged with centrifugal filter devices (10 000 MWCO, Amicon, Merck Millipore) into storage buffer. Purified M^pro^ was frozen in liquid nitrogen and stored at −80°C.

To produce proteins (Protein ID: P0C6X7) of different-length of the nsp7–10 region, BL21 Rosetta2 (Merck Millipore) were transformed, grown in culture flasks to 0.4–0.6 OD_600_ in 1 l, then induced with 50 µM anhydrotetracycline and continued to grow at 20°C overnight. To pellet, cultures were centrifuged (6000×***g*** for 20 min) and cells were frozen at −20°C. To separate soluble nsp's, pelleted cells were lysed in 1 : 5 (v/v) buffer B1 (40 mM phosphate buffer, 300 mM NaCl) with one freeze–thaw cycle, sonicated (micro tip, 70% power, 6 times on 10 s, off 60 s; Branson digital sonifier SFX 150) and then centrifuged (20 000×***g*** for 45 min). The proteins were isolated with Ni^2+^-NTA beads (Thermo Fisher Scientific) in gravity flow columns (Bio-Rad). First, the beads were equilibrated with 20 column volumes (CV) B1 + 20 mM imidazole, then bound to nsp's by incubation with crude extract for 60 min and finally to remove unspecifically bound proteins washed with 20 CV B1 + 20 mM imidazole followed by 10 CV of B1 + 50 mM imidazole. To elute the nsp's, eight fractions of 0.5 CV B1 + 300 mM imidazole were collected. Immediately after elution, the fractions were supplemented with 4 mM DTT. For quality analysis, SDS–PAGE was performed.

### FRET peptide assays

For SARS-CoV M^pro^ activity assays, Förster resonance energy transfer (FRET) peptide substrates (FPS) were commercially purchased (Eurogentec), designed as SARS-CoV cleavage site analogs of twelve amino-acid residues (P6–P6′) with a FRET pair labeling, namely the fluorophore (F) HiLyte488 at the N-terminus and the Quencher (Q) QXL520 at the C-terminus (Supplementary Tables S1 and S2).

Initially, the freeze-dried FPS were dissolved in DMSO to 2 mM and stored at −20°C, protected from light. For sample preparation, the peptides were serially diluted in 50 mM HEPES, 10% (v/v) glycerol, 1 mM DTT, pH 7.5 and final concentrations were adjusted by measuring A_280_ from five independent droplets, using *ε* = 73 000 mol^−1^ cm^−1^, the absorption coefficient of the HiLyte488 fluorophore as given by the manufacturer.

Assays were carried out using a 96-well plate reader (Infinite200, Tecan) with the following parameters: excitation wavelength 485 nm (bandwidth 9 nm), emission wavelength 535 nm (bandwidth 20 nm), gain 80, 10 flashes and 40 µs integration time. Furthermore, flat bottom black polystyrol 96-well plates (Greiner) were used for fluorescence applications.

Fluorescence coefficients *k*_sx_ and inner filter effect of the peptides were determined for each substrate according to previous protocols [33] (Supplementary Figure S1). The kinetic parameters were determined as suggested by Grum-Tokars et al. [34]. Briefly, 20 µl SARS-CoV M^pro^ (final conc. *c*_Mpro_ of 0.5 µM) was mixed with 80 µl FPS of six different concentrations (final conc. *c*_X,S_ of 2 µM, 1 µM, 0.5 µM, 0.25 µM, 0.125 µM and 0 µM) and fluorescence (AFU) was monitored every 30 s for 5 min. To calculate kinetic parameters, triplicate measurements were performed.

The fluorescence coefficients *k*_sx_ were used to convert the initial increase in AFU into the initial reaction rate, which was then normalized by *c*_Mpro_ to rate/[Enzyme]. For comparing substrate specificities, the rates/[Enzyme] were plotted against the substrate concentrations *c*_X,S_ resulting in process curves. The slopes of the process curves correspond to the apparent *k*_cat_/*K*_M_.

The influence of salt conditions on enzyme activity was determined by incubating 20 µl M^pro^ (final concentration 0.1 µM) with 80 µM FRET peptide substrate FPS4–5 (final concentration 1 µM) in varying NH_4_OAc (10% Glycerol, pH 7.5 and 50 mM, 165 mM, 280 mM, 395 mM, 510 mM, 625 mM, 740 mM, 855 mM, 970 mM, 1085 mM and 1200 mM) and NaCl (50 mM HEPES, 10% Glycerol, pH 7.5 and 0 mM, 120 mM, 240 mM, 360 mM, 480 mM, 600 mM, 720 mM, 840 mM, 960 mM, 1080 mM and 1200 mM). The fluorescence was followed every 60 s by monitoring AFU and initial slopes (AFU/s) were converted by the fluorescence coefficient *k*_sx_ into the initial rate (Supplementary Figure S2).

### SDS–PAGE

SDS–PAGE was performed with a 4%–12% gradient acrylamide Bis-tris gel with XT MES running buffer. For *in vitro* processing, 3.2 µM SARS-CoV M^pro^ was incubated with 13 µM SARS-CoV nsp7–9-His (ratio ∼1 : 4) at 4°C in 20 mM phosphate buffer, 150 mM NaCl, 1 mM DTT at pH 8.0.

### Native MS

To prepare samples for native MS measurements, they were buffer-exchanged into nanoESI-compatible solution. Protease M^pro^ was buffer-exchanged into 250–500 mM NH_4_OAc, 1 mM pH 8.0 by two cycles of centrifugal gel filtration (Biospin mini columns, 6000 MWCO, Bio-Rad). The nsp's were buffer-exchanged into 250–500 mM NH_4_OAc, 1 mM DTT, pH 8.0 by five rounds of dilution and concentration in centrifugal filter units (Amicon, 10 000 MWCO, Merck Millipore).

NanoESI capillaries were pulled in-house from borosilicate capillaries (1.2 mm outer diameter, 0.68 mm inner diameter, with filament, World Precision Instruments) with a micropipette puller (P-1000, Sutter Instruments) using a squared box ﬁlament (2.5 × 2.5 mm, Sutter Instruments) in a two-step program. Subsequently, capillaries were gold-coated using a sputter coater (Q150R, Quorum Technologies) with 40 mA, 200 s, tooling factor 2.3 and end bleed vacuum of 8 × 10^−2^ mbar argon.

Native MS was performed at a nanoESI quadrupole time-of-flight (Q-TOF) instrument (Q-TOF2, Micromass/Waters, MS Vision) modified for higher masses [35]. Samples were ionized in positive ion mode with voltages applied at the capillary of 1300–1500 V and at the cone of 130–135 V. The pressure in the source region was kept at 10 mbar throughout all native MS experiments. For the purpose of desolvation and dissociation, the pressure in the collision cell was adjusted to 1.3–1.5×10^−2^ mbar argon. Native mass spectra were obtained at an accelerating voltage of 10–30 V while for CID-MS/MS these voltages were increased to 30–200 V. In ESI-MS overview spectra for nsp's, the quadrupole profile was 1–10 000 *m/z*. In tandem MS, for precursor selection, LMres and HMres were adjusted at 10–30 V collisional voltage until a single peak was recorded, and then dissociation was induced.

To calibrate raw data, CsI (25 mg/ml) spectra were acquired and calibration was carried out with MassLynx (Waters) software. Data were analyzed using MassLynx (Waters) and Massign (by Morgner [36]). All determined masses are provided (Supplementary Table S3).

To start the processing reactions, nsp's and M^pro^ were mixed. Three independent reactions were started in parallel and incubated at 4°C. To acquire mass spectra at defined time-points, sample aliquots of 1–3 µl were taken by means of a microliter syringe (5 µl, Hamilton) with flexible fused silica tubing (Optronics) and loaded into in-house fabricated nanoESI capillaries, which were mounted on the nanoESI source, all within 2 min. Then, raw spectra were acquired in the first 300 scans (1 scan/s).

To analyze the data, the raw spectra were smoothed (2 × 5) in Masslynx 4.1 (Waters) and then nsp's were assigned to peak series. For each assigned mass species, signal responses (SRs), using the combined relative intensities of peaks, were summarized and normalized. This was done independently for each spectrum. Finally, the average and standard deviation for SR and time-points were calculated from three independent spectra. Poorly resolved spectra were not included in the data analysis.

To determine mass fractions (MFs), the SRs of assigned peaks for monomers and monomers in complexes were combined (Supplementary Table S4).

## Results

### Specific substrate efficiencies from FRET peptide assay

**Figure 1.**
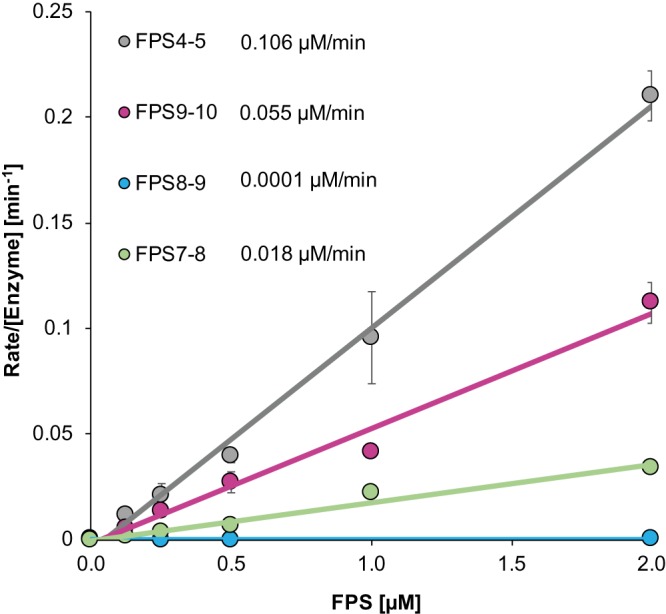
M^pro^ assay with FRET peptide substrates. Process curves of M^pro^ (0.5 µM) with different concentrations of FRET peptide substrates (FPS) in 50 mM HEPES, 10% Glycerol at pH 7.5. The slopes correspond to the apparent *k*_cat_/*K*_M_ as given in the legend. Standard deviations given in Supplementary Table S2.

Initially, the substrate specificity of SARS-CoV M^pro^ for cleavage of the nsp7–10 inter-domain junctions was studied by means of FPS's. The substrates FPS7–8, FPS8–9 and FPS9–10 represented the cleavage sites nsp7–8, nsp8–9 and nsp9–10, respectively. Additionally, FPS4–5 served as a positive control analogous to the highly efficient N-terminal auto-cleavage site nsp4–5 of SARS-CoV M^pro^ (nsp5)_._ The FPS's encompassed 12 amino-acid residues spanning positions P6 to P6′ around the cleavage site and were labeled with a fluorophore and a quencher at their N- and C-terminus, respectively (Supplementary Table S1). Substrate specificities were determined by monitoring the increasing fluorescence upon M^pro^ cleavage of the FPS's (Figure 1, Supplementary Figure S1 and Table S2). As expected, the assay showed FPS4–5 to be most efficiently cleaved amongst the FPS's tested here, with an apparent efficiency (*k*_cat_/*K*_M_) of 0.105 ± 0.005 µM^−1^ min^−1^. Compared with FPS4–5 (100%), substrates FPS7–8 (17.1%) and FPS9–10 (51.8%) were less efficiently cleaved while FPS8–9 (0.1%) was almost non-cleavable. The absolute and relative substrate efficiencies determined here agree with the results of similar experiments [20,34]. Considering that short peptides represent merely the primary structure of the cleavage site, more native-like substrates were prepared and tested to verify results obtained with these FPS's.

### Processing of nsp7–10

Next, we studied the substrate specificity of SARS-M^pro^ for folded proteins, which exhibit the structural environment of the cleavage sites beyond the amino-acid sequence. Therefore, full-length SARS-CoV His-nsp7–10 was recombinantly produced in *Escherichia coli* and purified. This protein construct had a non-cleavable N-terminal His-tag to reduce the number of cleavage product species and thereby simplified interpretation of the mass spectra.

**Figure 2.**
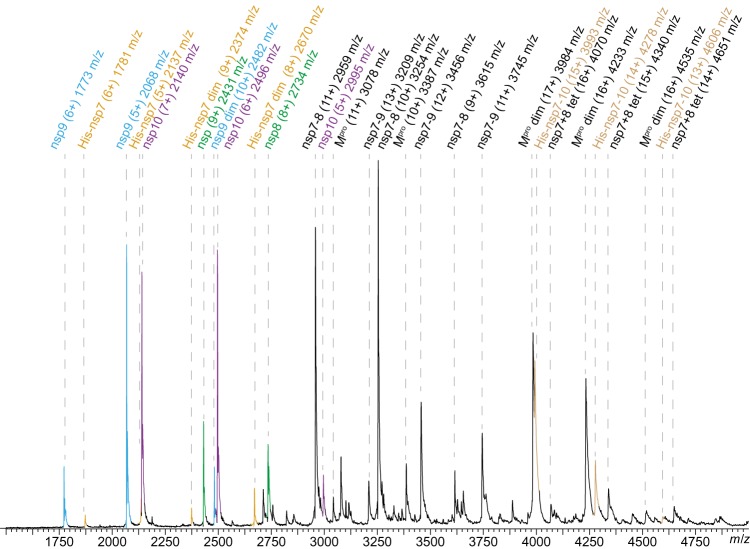
SARS-CoV nsp7–10 processing in native MS. Peak assignment in native MS after 6 h. Mass species, charge states and their *m/z* are labeled. For processing, 1.25 µM SARS-CoV M^pro^ and 12.5 µM SARS-CoV His-nsp7–10 (∼1 : 10 ratio) were incubated at 4°C in 250 mM NH_4_OAc, 1 mM DTT at pH 8.0. The components were mixed and after a defined incubation time, samples were injected into an electrospray capillary and mass spectra were acquired.

To monitor the processing dynamics, SARS-CoV His-nsp7–10 was cleaved by SARS-CoV M^pro^ in a test tube and samples were subjected to native MS. Cleavage was performed in a nanoESI compatible ammonium acetate (NH_4_OAc) solution at pH 8.0, which proved equally suitable to maintain protease activity compared with buffers with NaCl (Supplementary Figure S2). Processing products were observed as peaks in the mass spectra and assigned to protein mass species (Figure 2, Supplementary Table S3). When acquiring mass spectra at different times during processing, signals of three protein categories dominated successively, in the beginning, full-length nsp7–10 substrate and M^pro^ (∼1 h); subsequently, intermediate products (∼6 h), representing partially cleaved substrate; and in the end mature products encompassing nsp monomers and protein complexes (∼20 h) (Supplementary Figure S3). Covalent and non-covalent products were distinguished via CID-MS/MS.

**Figure 3.**
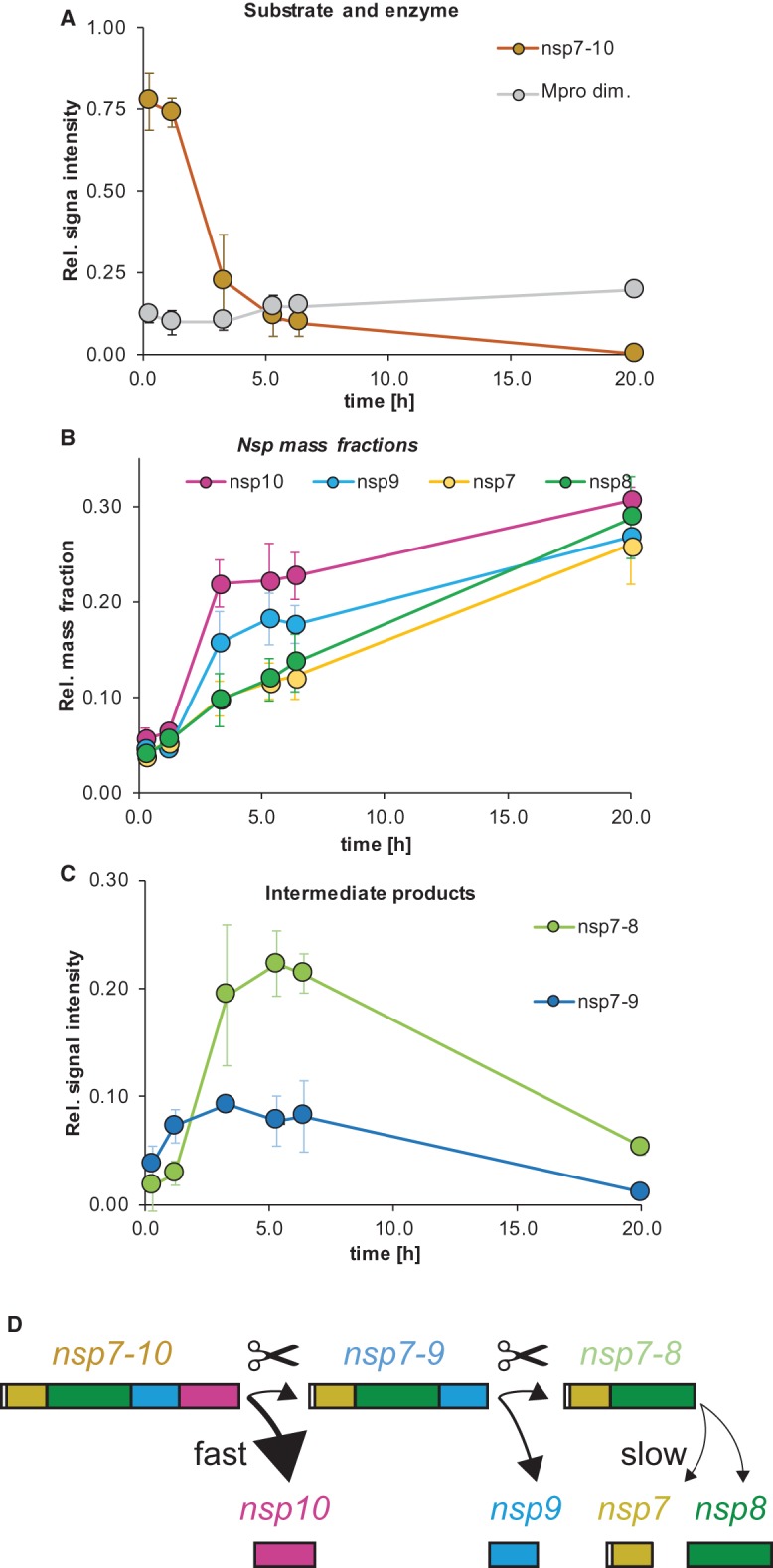
SARS-CoV nsp7–10 processing monitored by native MS: signal over time of protease, substrate and products. SARS-CoV nsp7–10 processing monitored by native MS: signal over time of protease, substrate and products. (**A**) Substrate and enzyme. (**B**) Mass fractions over time show the order of nsp release. (**C**) Intermediate products. Error bars depict standard deviation (*N *= 3). Time points (AVG ± SD, *N *= 3): 0.25 ± 0.1 h, 1.2 ± 0.1 h, 3.3 ± 0.2 h, 5.3 ± 0.3 h, 6.4 ± 0.3 h and 20 ± 0.5 h. (**D**) Schematic illustration of cleavage order and efficiency.

The order of reactions became evident from monitoring peak intensities over time. The relative signal intensity of M^pro^ remained stable while nsp7–10 decreased (Figure 3A), indicating that M^pro^ depleted nsp7–10. After 20 h, no signal of nsp7–10 was detected anymore and therefore, cleavage was considered complete.

To obtain the sequence of nsp release, the ratio of the mature nsp monomers was followed over time. However, some non-covalent interactions of the nsp's introduced a bias to the relative peak intensities among monomers (Supplementary Figure S4). To correct for this bias, signal intensities were converted into MFs, which include the relative signal of each released subunit over time (Supplementary Table S4). Thereby, MFs demonstrated a processing order of the nsp9–10 site first, the nsp8–9 site second, and the nsp7–8 site third (Figure 3B). MFs were similar at the end-point of processing (∼20 h) showing that the individual nsp's were all cleaved off nsp7–10 (Supplementary Figure S4).

To determine the relative cleavage efficiencies in the full-length substrate, we used the relative MFs between nsp10, nsp9, nsp8 and nsp7 after 3.3 h (100%, 66%, 36% and 36%, respectively). These values suggest decreasing cleavage efficiency from the C- to the N-terminus of SARS nsp7–10. Consistently, the cleavage pattern of the intermediate products revealed that nsp7–10 is predominantly cleaved to nsp7–9 and only then to nsp7–8 (Figure 3C). The time-course of processing of the SARS-CoV full-length substrate as determined here via native MS corresponded well with results from SDS–PAGE using the shorter SARS-CoV His-nsp7–9 substrate (Figure 3C, Supplementary Figure S5). Additionally, shorter substrates were also analyzed in native MS, fully in line with the results obtained for SARS-CoV nsp7–10 (Supplementary Figure S6).

The results from FRET peptide assay and full-length protein cleavage are in conflict regarding the suggested cleavage order. This is particularly interesting for nsp8–9, because of its *Coronaviridae*-wide conserved NNE motif at P1′–P3′. Previously, it was suggested that this motif could confer poor substrate properties to the nsp8–9 cleavage site, which would result in a considerable influence on processing order and RTC assembly [22]. In fact, two observations made by other researchers provide an indication for a peptide-specific effect: Fan et al. analyzed the secondary structure of nsp peptides and observed that nsp8–9, comprising the NNE motif and closely resembling the FPS8–9 tested here, had a higher propensity for α-helices than other nsp peptides [37,38]. Moreover, Chuck et al*.* [39] reported that β-strands in substrates promote binding to M^pro^. The results shown in our work indicate that the structural layout of the cleavage site is different when comparing peptide to the full-length protein, which plays a role in endowing the nsp8–9 cleavage site with decent cleavability. Since the full-length protein possesses the structural environment of the cleavage sites, we consider results with this substrate more relevant for studying the processing pattern of the CoV polyprotein.

### Protein complex formation upon processing of nsp7–10

In parallel, we applied CID-MS/MS to identify non-covalent interactions. Thereby, covalent processing intermediates were distinguished from newly formed complexes. For instance, nsp7–8 intermediate and nsp7+8(1 : 1) dimer overlap in mass spectra (Supplementary Figure S7). Most importantly, we identified an nsp7+8 (2 : 2) complex, undescribed before, which is subject to detailed analysis later on. Here, this complex’ intensity in native MS remained low, most likely due to interference by the non-cleavable His-tag N-terminal of nsp7. Furthermore, homo-dimers of nsp7 and nsp9 were found (Supplementary Figure S8). The MFs of nsp9 monomers and dimers (AVG ± SD, *N* = 3; 75 ± 11% and 25 ± 2%, respectively) were determined, based on the intensity ratio of the assigned peaks. Although the X-ray structure of nsp9 was reported as a dimer, its low abundance found here is consistent with a low dimer affinity in solution [40]. So far, nsp7's ability of dimerization in the absence of other proteins has already been noted, but no function has been suggested [27] and therefore, more detailed information remains elusive. Many interactions between components of nsp7–10 have been reported in the past, however, nsp interactions other than those reported above were completely absent, despite the high sensitivity of native MS.

### Complex formation of nsp7+8

**Figure 4.**
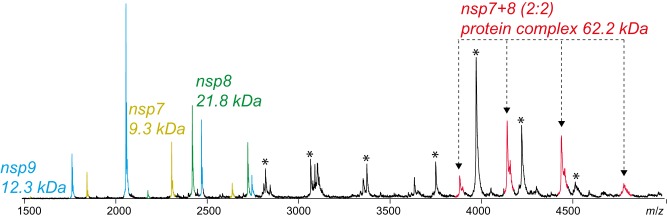
SARS-CoV nsp7+8 forms a hetero-tetramer. Upon cleavage of nsp7–9-His, a putative nsp7+8 (2 : 2) tetramer (red) emerged. Other assigned peaks correspond to M^pro^ (asterisk), nsp7 (yellow), nsp8 (green) and nsp9 (blue). For cleaving the precursor, 2 µM SARS-CoV M^pro^ was incubated with 14 µM SARS-CoV nsp7–9-His (ratio ∼1 : 7) at 4°C in 300 mM NH_4_OAc, 1 mM DTT at pH 8.0 overnight.

**Figure 5.**
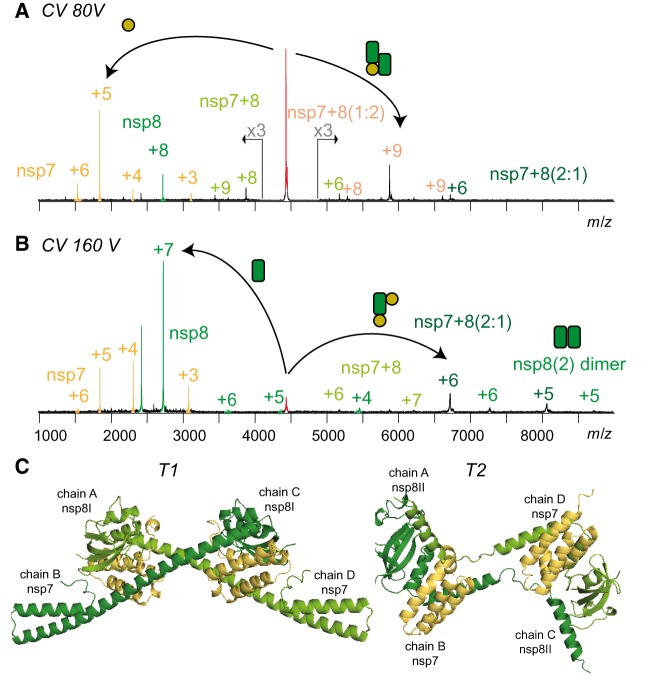
CID-MS/MS reveals the architecture of the nsp7+8 complex. Product ion spectra showing two CID dissociation pathways of SARS-CoV nsp7+8 hetero-tetramer (+14, 4449 *m/z*) at (**A**) 50 V and (**B**) 80 V collisional energy. (**C**) Two conformers denoted as T1 and T2 extracted from a hexa-decameric SARS-CoV nsp7+8 (PDB 2AHM) [29] and both in line with the native MS results.

Finally, the complex of nsp7+8 was investigated in detail. To promote interactions of nsp's with authentic amino-acid sequence, we engineered a SARS-CoV nsp7–9-His containing a cleavable His-tag at the C-terminus. Upon processing of this substrate by M^pro^, we tested the landscape of mass species via native MS. Peak envelopes were detected for the monomeric products nsp7, nsp8 and nsp9 as well as for M^pro^ monomer and dimer. While nsp9 did not interact with any of the other subunits even after cleavage of the tag, nsp7+8 formed a hetero-tetramer of nsp7+8(2 : 2) (62.2 kDa) (Figure 4). This stoichiometry was predicted based on the molecular weights of the subunits (2×9.3 kDa + 2×21.8 kDa = 62.2 kDa). However, for further evidence, molecular ions of three different charge states of the putative nsp7+8 hetero-tetramer (4449 *m/z*, +14; 4153 *m/z*, +15; 2893 *m/z*, +16) (Figure 5A) were selected as precursor and subjected to CID-MS/MS. To successively induce subunit dissociation, we increased the collision voltage (CV) stepwise. Thereby, the overall dissociation pattern was exposed (Figure 5A and Supplementary Figure S9), which confirmed the hetero-tetrameric (2 : 2) complex stoichiometry.

Two alternative dissociation pathways revealed the arrangement of subunits within the complex (Figure 5B). The hetero-tetramer dissociated at lower activation energy into nsp7 and nsp7+8(1 : 2), and alternatively, at elevated collisional energy into nsp8 and nsp7+8(2 : 1). Additionally, the product ions nsp7+8(1 : 1) and nsp8(2) provided evidence that their subunits interacted in the complex. The alternative dissociation routes may have resulted from analogous energy absorption paths, either due to similar binding contribution of nsp7 and nsp8 in the complex or due to different gas-phase conformers.

## Discussion

Here, we analyzed the processing of the SARS-CoV polyprotein region nsp7–10 *in vitro*. The mechanistic system of processing resembles a limited proteolysis of a folded protein, and we used native MS to monitor the covalent and non-covalent products. Thereby, we gained insights into the coordinated release of nsp monomers, intermediate products and the formation of complexes. A relatively long-lived nsp7–8 product and a short-lived nsp8–9 intermediate product were observed, which were not expected based on protease activities measured on isolated peptide substrates. Therefore, we conclude that the processing of nsp7–10 is not only coordinated by the amino-acid sequence itself but also the structural environment of the nsp inter-domain junctions. This, in turn, may lead to different processing intermediates than expected based on results from protease assays at isolated peptides. To collect evidence of such intermediates *in vivo* appears challenging by more traditional techniques. To reveal their relevance in the viral life-cycle, novel MS techniques may be advantageous, e.g. single-cell proteomics and *in vivo* cross-linking MS [41,42].

The major identified protein complex was a hetero-tetramer of nsp7+8 (2 : 2). Although, molecular structures of SARS-CoV nsp7+8 have been reported as dimer (1 : 1) containing N-terminally chopped nsp8 and as a hexa-decamer (8 : 8) [26,29] up to date, a hetero-tetrameric interaction has not been shown. In terms of stoichiometry and subunit connectivity, only nsp7+8(2 : 2) subcomplexes of the hexa-decamer resemble our results [29]. Specifically, in two conformers, T1 and T2 (Figure 5C), nsp8 builds a scaffold with head-to-tail interaction and nsp7 subunits sandwich the scaffold without self-interaction. All interfaces are mainly hydrophobic. However, three polar bonds within or in the proximity of the nsp8 shaft domain facilitate binding to nsp7. Polar bonds more readily persist in the gas phase than hydrophobic interactions, which explains higher gas-phase stability for nsp7+8(1 : 1) over nsp8(2). The total buried surface areas of T1 and T2 are virtually equal (Supplementary Table S5). Nevertheless, the nsp8(2) dimer scaffold in T2 is compact because a loop within the N-terminal α-helix allows bending and interacting with the other nsp8, whereas in T1 the α-helix extends to interact with another tetramer in the crystal lattice. Hence, T2 represents a more likely structure of nsp7+8(2 : 2) in solution.

Finding a link between the plasticity of nsp7+8 and its defined functions, particularly in the context of the CoV-RTC, remains to be addressed. This understanding was furthered by the identification of the SARS-CoV RdRp complex as nsp(7+8)+8+12 ((1: 1) : 1 : 1) [28]. However, in this complex, the stoichiometry of nsp7+8 is not consistent with the quaternary structures that have been previously reported or ours presented here [27,29]. Nevertheless, the quaternary structure of the nsp7+8 complex in the absence of other RTC subunits could be different and may be an important intermediate for either stability, functionality or subsequent interactions in solution.

In summary, we performed a comprehensive analysis of specific processing and complex formation of a folded viral polyprotein with native MS. While it remains a challenge to perform quantitative MS from heterogeneous protein mixtures, the usage of MFs disentangled the mass species and allowed following the processing dynamics. Intriguingly, our results suggest that the processing pattern of SARS-CoV nsp7–10 could have evolved to tailor a remaining nsp7–8 intermediate product. This intermediate product may have an independent enzymatic function [27] but finally serves as an efficient precursor for nsp7+8(2 : 2) hetero-tetramer formation, as stimulant of the CoV RdRp [24]. Extending our approach to processing of different CoV species and longer polyproteins will increase understanding of the formation of the functional CoV-RTC.

## Abbreviations

CID: collision-induced dissociation
CV: collision voltage
FPS: FRET peptide substrates
FRET: Förster resonance energy transfer
MFs: mass fractions
MS: mass spectrometry
nanoESI: nano-electrospray ionization
RdRp: RNA-dependent RNA-polymerase
RTC: replication/transcription complex
SARS-CoV: severe acute respiratory syndrome coronavirus
SRs: signal responses
